# Combinatorial Use of Both Epigenetic and Non-Epigenetic Mechanisms to Efficiently Reactivate HIV Latency

**DOI:** 10.3390/ijms22073697

**Published:** 2021-04-02

**Authors:** Joseph Hokello, Adhikarimayum Lakhikumar Sharma, Mudit Tyagi

**Affiliations:** 1 Department of Basic Science, Faculty of Science and Technology, Kampala International University-Western Campus, Bushenyi P.O. Box 71, Uganda; hokello.joseph@kiu.ac.ug; 2 Center for Translational Medicine, Thomas Jefferson University, 1020 Locust Street, Philadelphia, PA 19107, USA; LakhikumarSharma.Adhikarimayum@jefferson.edu

**Keywords:** epigenetic, non-epigenetic, HIV latency, reactivation, transcription

## Abstract

The persistence of latent HIV provirus pools in different resting CD4+ cell subsets remains the greatest obstacle in the current efforts to treat and cure HIV infection. Recent efforts to purge out latently infected memory CD4+ T-cells using latency-reversing agents have failed in clinical trials. This review discusses the epigenetic and non-epigenetic mechanisms of HIV latency control, major limitations of the current approaches of using latency-reversing agents to reactivate HIV latency in resting CD4+ T-cells, and potential solutions to these limitations.

## 1. Introduction

When our immune system encounters a foreign body, such as an antigen or allergen, the naïve CD4+ T-cells become stimulated or metabolically activated. Upon antigenic stimulation, naïve CD4+ T-cells undergo clonal proliferation and transformation to become effector CD4+ T-cells. Upon fulfillment of their effector functions, most of the effector CD4+ T-cells undergo programmed cell death, also known as apoptosis. However, a minuscule fraction of the effector CD4+ T-cells reverts into quiescence to become resting memory CD4+ T-cells with a half-life that can last a lifetime. Resting memory CD4+ T-cells are antigen-specific and, thus, mediate immunologic memory such that following a subsequent encounter with the same antigen, they can mount a quick and vast immune response.

As opposed to naïve CD4+ T-cells, metabolically active CD4+ T-cells are required for efficient HIV infection and replication [1,2]. Upon successful infection of individual activated target CD4+ T-cells, the majority of the infecting viruses undergo functional decay before or during the reverse transcription step [3]. Metabolically active effector CD4+ T-cells are the major population functionally infected by HIV, due to the availability of required metabolites for HIV life cycle. Although the immune system can temporarily clear the HIV antigens in the peripheral circulation, it is unable to completely control/eradicate HIV infection. Additionally, upon temporal clearance of the HIV antigens in the blood, a certain population of effector CD4+ T-cells revert back and become quiescent resting memory CD4+ T-cells. Due to the quiescent or metabolically silent nature of resting memory CD4+ T-cells, HIV is unable to go through its life cycle and becomes transcriptionally silent or latent. Thus, HIV latency is a condition where quiescent resting memory CD4+ T-cells harbor transcriptionally silent HIV proviruses. Most of the latent or hibernating HIV proviruses are capable of entering into a fully productive lytic infection but await optimal conditions, that usually arise following the reactivation of quiescent memory CD4+ T-cells, which again make them metabolically active. However, the molecular control of HIV latency in resting memory CD4+ T-cells is multi-pronged [4,5], and effective and efficient reactivation of the latent HIV proviruses in all subsets of quiescent resting memory CD4+ T-cells requires a combinatorial approach involving both epigenetic and non-epigenetic mechanisms of HIV latency reactivation.

## 2. Epigenetic Control of HIV Latency in Resting Memory CD4+ T-cells

Molecular control and maintenance of HIV latency are mediated by multiple factors acting in concert, including epigenetic and non-epigenetic mechanisms [4,5]. HIV integration preferentially occurs within actively transcribed cellular genes [6]. Upon provirus integration, an ordered nucleosomal structure is assembled within the HIV long terminal repeat (LTR) which functions as the HIV promoter [7,8,9]. The nucleosomal structure, particularly nucleosome-1 (Nuc-1), positioned around the HIV transcription initiation start site, contributes to HIV latency or transcriptional silencing by blocking RNA polymerase II (RNAP II) initiation of transcription [7].

In addition, other epigenetic mechanisms mediating HIV latency include histone deacetylation [10], histone methylation [11], and deoxyribonucleic acid (DNA) methylation [12,13]. Epigenetic modification of the HIV LTR through histone deacetylation is mediated by the recruitment of histone deacetylases (HDACs). In this regard, Margolis and his group demonstrated cooperative binding of Late SV40 factors (LSF) and yin-yang 1 (YY1) to the HIV LTR, which mediated subsequent recruitment of HDAC1 to the Nuc-1 region of the HIV promoter [10,14,15]. Similarly, Williams et al. [16] demonstrated that NF-κB p50 subunits are constitutively bound to the HIV LTR promoter of transcriptionally silent HIV proviruses, which mediate HDAC1 recruitment to the HIV LTR, resulting in HIV transcriptional repression. Furthermore, Tyagi et al. demonstrated that the C-promoter binding factor 1 (CBF-1), which is a key regulator of the Notch signaling pathway, also facilitates HIV latency by recruiting HDACs to the HIV LTR [17,18]. To extend those observations further, Tyagi group recently, in Sharma et al. [19], demonstrated that CBF-1, by recruiting the polycomb repressive complexes PRC1 and PRC2 to LTR, facilitates both the establishment and the maintenance of HIV latency.

Epigenetic control of HIV-1 latency is also mediated through histone methylation. In this case, methylated histone H3 is trimethylated on either histone lysine 9 (H3K9me3) or histone lysine 27 (H3K27me3) [20,21,22] or dimethylated on lysine 9 (H3K9me2) [23]. All these modifications are repressive markers for HIV gene expression. Epigenetic silencing of HIV-1 LTR through histone deacetylation and histone methylation results in multifaceted, heterogeneous patterns of DNA methylation along the HIV proviral genome [12,13]. Complex epigenetic heterogeneity was found to exist among clonal populations of CD4+ T-cells, despite carrying identical integrated proviruses [12,13]. Conceivably, this accounts for variations that exist between different subsets of silenced proviruses which reactivate differently in response to exogenous signals and most latency-reversing agents (LRAs).

## 3. Non-Epigenetic Regulation and Reactivation of HIV Latency by NF-κB and P-TEFb

Unlike in quiescent resting memory CD4+ T-cells, HIV potently replicates in activated CD4+ T-cells. In latently infected resting memory CD4+ T-cells, transcription factors such as the nuclear factor kappaB (NF-κB), nuclear factor of activated T-cells (NFAT), and activator protein 1 (AP-1) are all sequestered in the cytoplasm but soon translocate into the nucleus following T-cell stimulation. Multiple signaling pathways, including T-cell receptor (TCR) activation [24,25,26,27] or cytokine stimulation [20,28] that are capable of inducing NF-κB or NFAT, are able to potently induce HIV proviral transcription.

The nuclear induction of NF-κB and its binding to the HIV LTR promoter trigger HIV proviral transcription by recruiting histone acetyltransferases (HATs) to the HIV promoter [29,30,31,32]. Histone acetylation at the HIV promoter subsequently results in the recruitment of the chromatin remodeling complex BAF, which leads to transcriptional activation by displacing Nuc-1, positioned immediately downstream of the transcription start site [7,8,9,33,34,35]. Conceivably, the mode of HIV transcription initiation mediated by NF-κB is also mediated by NFAT transcription factors. Paradoxically, using 2D10 Jurkat T-cell clones we [36] most recently demonstrated that while NF-κB is able to activate HIV latency, NFAT inhibits HIV LTR transcription through competitive binding to the overlapping NF-κB binding sites within the HIV LTR following T-cell receptor activation.

There is a low level of viral Tat protein in latently infected quiescent resting memory CD4+ T-cells due to restricted ongoing HIV transcription in these cells. However, cellular activation results in nuclear translocation of NF-κB, which initiates HIV transcription leading to an increase in viral Tat protein levels. Viral Tat protein functions by unusually binding to the transcription response (TAR) element found in nascent mRNA. TAR is a stem–loop RNA structure located at the 5′ end of all viral transcripts. Viral Tat binds to TAR and recruits positive transcription elongation factor b (P-TEFb), which is a cellular transcription elongation factor [37]. The cyclin-dependent kinase 9 (CDK9) subunit of P-TEFb then phosphorylates the C-terminal domain (CTD) of the largest subunit of RNAP II, leading to the enhancement of HIV transcription elongation [37,38]. Using the clone 2D10 model system of HIV latency, Pearson et al. [20] demonstrated that, indeed, reactivation of HIV latency is strictly dependent on NF-κB and viral Tat protein in Jurkat T-cells. Subsequently, Tyagi et al. [17] demonstrated that HIV latency, in primary T cells, is restricted to both initiation and elongation phases. Therefore, in order to reactivate latent HIV provirus in primary T cells, there is a need to activate both NF-κb and P-TEFb. Hence, restricted nuclear levels of P-TEFb in latently infected primary CD4+ T-cells strongly prohibited HIV transcription even when NF-κB was induced by TNF-α stimulation. Tyagi et al. found that TCR activation, which proficiently induces transcription factors including NF-κB through the protein kinase C pathway and P-TEFb, mobilized through an ERK-dependent pathway [26], was able to efficiently reactivate latent HIV in primary CD4+ T -cells [17]. Recently, Hokello et al. [36] demonstrated that AP-1 synergizes with NF-κB to modulate HIV transcriptional elongation following TCR activation. Initially, Tyagi et al. [39] demonstrated a functional interaction between DNA-dependent protein kinase (DNA-PK) and RNAP II during HIV transcription, such that the knockdown of endogenous DNA-PK using small hairpin RNAs resulted in a significant reduction in HIV transcription. Recently, the Tyagi lab [40] specifically showed that DNA-PK, besides catalyzing RNAP II CTD phosphorylation, also enhances the recruitment of P-TEFb to the HIV LTR. Thus, DNA-PK concomitantly increases the phosphorylation of the CTD of RNAP II at Serine 5 and Serine 2, thereby stimulating both HIV transcriptional initiation and elongation. The Tyagi lab also demonstrated that DNA-PK promotes the release of paused RNAP II through the phosphorylation of tripartite motif-containing 28 (TRIM28) at the HIV LTR [40]. These results demonstrate that DNA-PK participates at multiple levels in order to facilitate HIV transcription.

## 4. Nuclear Factor kappaB (NF-κB) Transcription Factors

Different biological functions such as the innate and adaptive immune responses are controlled by NF-κBs, which are a superfamily of DNA-binding transcription factors [41,42]. Alternatively, referred to as Rel transcription factors, NF-κBs consist of five members, all of which have the conserved N-terminal Rel homology domain (RHD). The RHD consists of both the DNA-binding and the NF-κB dimerization domains. The nuclear localization sequence (NLS) is also located within the RHD. Among the NF-κB family members, p50 and p52 lack a transcription activation domain (TAD), while p65, also known as RelA, RelB, and c-Re,l contain a TAD and, for this reason, p50 and p52 are unable to activate transcription on their own [43]. Interestingly, NF-κBs can form functional hetero- or homodimers within their own members, and the most abundant heterodimers are formed between p65 and p50, which also happen to be the most transcriptionally active form, although homodimers of p65 are also known to potently activate transcription [43]. Heterodimers of p50 and p52 or their homodimers are unable to activate transcription, due to the fact that p50 and p52 lack a TAD. The RHD comprises two independently folded immunoglobulin-like sub-domains referred to as RHR-N and RHR-C. Rel homology region N is used in the binding of specific DNA sequences, while RHR-C mediates NF-κB dimerization and binding to IkB-α inhibitor [41,44]. IkBs contain ankyrin repeats at the N-terminus and a PEST motif at the C-terminus. The major form and the best-studied IkBs is IkB-α, which binds NF-κB dimers and blocks their NLS, while causing their sequestration in the cytoplasm [41].

However, TCR stimulation results in the activation of the canonical pathway of NF-κB induction, whereby the catalytic subunit IKK-β, together with the regulatory subunit IKK-γ or NEMO, becomes activated and phosphorylates IkB-α leading to its ubiquitination, proteasomal degradation, and the resultant nuclear localization of NF-κB to regulate the expression of NF-κB-responsive genes [41]. In addition to the TCR signals, other proinflammatory cytokine signals are able to induce nuclear translocation of NF-κB, including signaling through tumor necrosis factor-apha (TNF-α) receptor and interleukin 1 (IL-1) receptor. Similarly, a variety of pathogen-associated molecular patterns (PAMPs) involving toll-like receptors (TLRs) such as TLR-2, TLR-4, and TLR-7 can induce NF-κB nuclear mobilization and regulate NF-κB-responsive genes [45,46,47]. Though some studies have demonstrated that these cytokine signals and TLR signals can independently reactivate HIV latency in some cellular models of HIV latency [45], Tyagi et al. [17] exhaustively demonstrated that, unlike TCR activation, TNF-α stimulation of latently infected primary CD4+ T-cells to induce nuclear NF-κB failed to activate HIV transcription due to nuclear restriction of P-TEFb, which is not induced by TNF-α stimulation. This observation is clear evidence that any non-epigenetic approach that is employed to deplete latently infected resting memory CD4+ T-cells should be able to cause nuclear mobilization of not only NF-κB but also P-TEFb. Other than exogenous extracellular signals, several intracellular agonists have been demonstrated to induce NF-κB nuclear translocation, and these include PKC agonists such as prostatin, bryostatin, ingenol, and phorbol myristic acid (PMA) [26,48]. Interestingly, these PKC agonists are also able to potently induce P-TEFb nuclear mobilization.

## 5. Positive Transcription Elongation Factor b (P-TEFb)

**Figure 1:**
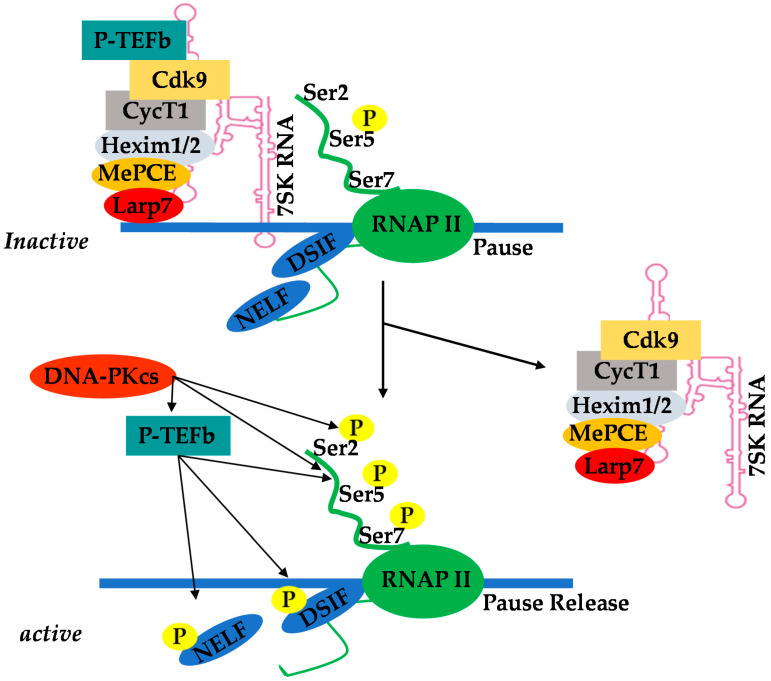
Equilibrium of positive transcription elongation factor b (P-TEFb) between the active pool and the inactive complex of 7SK small nuclear ribonucleoprotein (7SK snRNP).

Positive transcription elongation factor b (P-TEFb), originally identified as a general transcription factor that stimulates RNAP II transcriptional elongation, was subsequently discovered to be an essential cellular co-factor of HIV transcription mediated by viral Tat proteins [49]. In Jurkat T-cells, which are actively replicating cells, P-TEFb exists in an active pool, either by itself or in association with various proteins that recruit P-TEFb to its target genes where RNAP II is engaged (free pools of P-TEFb), and in an inactive complex comprising hexamethylene bisacetamide-inducible mRNAs 1 and 2 (HEXIM1/2) proteins, La-related protein 7 (LARP7), and methyl phosphate capping enzyme (MePCE), also referred to as 7SK small nuclear ribonucleoprotein (7SK snRNP) [50,51,52,53,54,55,56,57,58,59]. In Jurkat T cells, about 50% to 90% of P-TEFb is found in the 7SK snRNP inactive complex (Figure 1). This P-TEFb equilibrium maintains the level of active P-TEFb that stimulates transcription elongation.

The recruitment of P-TEFb upon cellular activation is a key checkpoint of RNAP II pause–release and subsequent induction of transcription elongation [60,61,62]. Chromatin-binding proteins such as bromodomain-containing protein 4 (Brd4) are P-TEFb binding factors that recruit it to its target genes [63,64]. However, in the case of HIV transcription, P-TEFb is recruited by viral Tat protein. The sequestered P-TEFb in the 7SK snRNP complex effectively prevents basal transcriptional activation by Tat-independent recruitment of P-TEFb to the provirus. Thus, Tat overcomes this barrier by disrupting the 7SK snRNP complex thorugh competition with HEXIM for CycT1 binding [65,66,67]. A study suggests that cyclin T1 acetylation also triggers dissociation of HEXIM1 and 7SK snRNA from the inactive 7SK snRNP complex and activates the transcriptional activity of P-TEFb [68].

Because of the highly restricted levels of cyclin T1 in primary resting memory CD4+ T-cells [17,69] and primary monocytes [70], activation of P-TEFb in these cells requires multiple steps involving both the initial assembly of the 7SK snRNP complex and its relocalization to nuclear speckles before it becomes available and accessible to Tat and the other transcription machinery. Another important observation to consider is that compounds such as bromodomain and extraterminal inhibitors (BETi) also target P-TEFb [71,72,73]. When cells are treated with BETi, such as JQ1 and I-BET, the Brd4/P–TEFb complex bound to histone H3 acetylated at the lysine residue 27 (H3K27Ac) soon dissociates from transcriptionally active chromatin, suppressing P-TEFb-dependent transcription of genes associated with the transcriptionally active modified histone H3K27Ac [73]. Similarly, BETi treatment releases P-TEFb from its inactive pools in 7SK snRNP to activate P-TEFb-dependent transcription [50]. However, this P-TEFb release also triggers HEXIM1 upregulation and concomitant suppression of P-TEFb-dependent transcription by forming the 7SK snRNP complex [74]. Therefore, the overall effects on cellular gene expression resulting from BETi are a mixture of activation and suppression of P-TEFb-dependent transcription, which is also dependent on particular genes, chromatin structure, cellular status, and cell type, among other factors. Therefore, this suggests that the use of BETi such as JQ1 to reverse HIV latency may be unsustainable.

## 6. Limitations to the Current Use of Latency-Reversing Agents

Recent studies of HIV latency tested novel therapeutic approaches to deplete the latent HIV provirus pools in the peripheral circulation [75,76,77,78,79,80]. For instance, the “shock and kill” strategy has undergone several clinical trials and utilizes latency-reversing agents (LRAs) or transactivators, which are given to patients on highly active antiretroviral therapy (HAART) in order to reactivate transcription of the latent HIV proviruses in latently infected resting memory CD4+ T-cells [81]. The “shock and kill” strategy aims to limit the exposure to LRAs until the latent provirus pools are reactivated to an extent that HAART can be discontinued without the risk of viral rebound. The most common LRAs employed in the “shock and kill” strategy include histone deacetylase inhibitors (HDACi) and protein kinase-C (PKC) agonists. The major aim of the ‘shock and kill’ strategy is to activate HIV-1 transcription such that activated latently infected resting memory CD4+ T-cells can be cleared from the peripheral circulation by the viral cytopathic effects and/or host cytolytic immune effectors, while at the same time limiting new target cell infection through HAART [81]. The “shock and kill” strategy initially appeared promising as the ultimate solution to HIV-1 eradication; however, following over 15 clinical trials that tested LRAs of distinct mechanistic classes [82,83], the results were not encouraging, since only limited reactivation of the latent HIV reservoirs was achieved. Likewise, ex vivo experiments that tested potent single LRA regimens using aviremic patient cells demonstrated that viral reactivation occurs for only a miniscule fraction of latently infected cells [84,85,86].

Testing of LRAs from different mechanistic classes in clinical trials was informed by encouraging results and recommendations from several studies which demonstrated that the use of LRAs alone or in combinations, such as of PKC agonists which induce NF-κB with those that induce P-TEFb, potently induced latent HIV provirus reactivation both in in vitro and ex vivio experiments [48,87].

One of the major limitations of using LRAs is that they were intended only to target circulating latently infected CD4+ T-cells, and yet, the majority of latently infected HIV provirus pools are found in anatomical sites, where LRAs access may be highly restricted. The “shock and kill” strategy requires that activated virus-expressing CD4+ T-cells are cleared by cytotoxic T-lymphocytes (CTLs), which may also need to be boosted due to the fact that during the chronic phase of HIV infection, the cytolytic capacity of CD8+ T-cells is greatly impaired and not restored by HAART.

The use of individual LRAs alone has failed to reduce the size of the latent provirus pools, majorly because of the heterogeneity of the latent HIV reservoirs [88]. Because HIV latency occurs in different cell types in different anatomical sites, there is, therefore, a need to first understand the mechanisms of HIV latency control and reactivations in these cellular and tissue reservoirs. For instance, the latently infected CD4+ T-cell reservoirs are so diverse and can be distinguished by their state of differentiation or functions [88]. Considering the differentiation state, there are naïve CD4+ T-cells, which are, however, rarely infected by HIV, and memory CD4+ T-cells. Latently infected memory CD4+ T-cells can be further grouped into four subsets, namely, central memory CD4+ T-cells (TCM), effector memory CD4+ T-cells (TEM), transitional memory CD4+ T-cells (TTM), and stem cell-like memory CD4+ T-cells (TSCM). Resting memory CD4+ T-cells constitute the vast majority of latent HIV provirus pools in different anatomical sites. The chromosomal environment of the integrated HIV proviruses is reported to interfere with HIV latency; however, it is still unclear whether the molecular mechanisms of HIV latency control are the same in all the different latently infected resting memory CD4+ T-cell subsets. Considering these limitations of LRAs, additional strategies to deplete latently infected resting memory CD4+ T-cell subsets are urgently needed.

## 7. Combinatorial Use of LRAs That Utilize Both Epigenetic and Non-Epigenetic Mechanisms to Reactivate HIV Latency in Resting Memory CD4+ T-Cell Subsets

Studies that have attempted to address the problems of HIV latency reversal in different memory CD4+ T-cell subsets analyzed integration sites data in different in vitro models of HIV latency that are based on primary CD4+ T-cells or T-cell lines [89]. In this case, latent HIV proviruses were analyzed based on proviral expression status versus genomic features. Results of this analysis demonstrated that genomic features were significantly associated with HIV proviral expression ability across different individual latency models. Most recently, Pardons et al. [90] analyzed the levels of cellular factors involved in HIV gene expression in TCM, TTM, and TEM memory CD4+ T-cell subsets. In particular, the levels of acetylated histones H3, active NF-κB, and active P-TEFb were measured in these memory CD4+ T-cell subsets following treatment with different LRAs. Interestingly, Pardons et al. observed that Vorinostat and Romidepsin displayed opposite abilities to induce histone acetylation across the different memory CD4+ T-cell subsets. Whereas PKC agonists effectively induced NF-κB activation through phosphorylation in TCM cells, they potently activated P-TEFb in TEM cell subsets. Furthermore, while ingenol, which is a PKC agonist, displayed modest activities in all memory CD4+ T-cell subsets, a combination of ingenol and HDACi dramatically increased HIV latency reactivation across all the memory CD4+ T-cell subsets.

Similarly, Grau-Exposito et al. [91] demonstrated the inability of different potent LRAs to induce HIV proviral expression across all the different memory CD4+T-cell subsets, even when LRAs of different mechanistic classes were combined, provided the LRAs were all HDACi. On the contrary, a combination of panobinostat, HDACi, and bryostatin, a PKC agonist, was most effective in reactivating HIV expression in all different memory CD4+ T-cell subsets. These observations suggest that cellular reservoirs of HIV latency respond differently to different LRAs when used alone, such that a combinatorial approach involving the use of LRAs that utilize epigenetic and non-epigenetic mechanisms to reactivate HIV latency is critically required in order to effectively reactivate HIV latency in all subsets of memory CD4+ T-cells. The PKC agonists including prostatin, bryostatin, and ingenol are known to effectively activate both NF-κB and P-TEFb [48].

## 8. Conclusions

The existence of latent HIV provirus pools remains a major barrier to successful HIV treatment and eradication. LRAs belonging to classes of agents that target different mechanisms involved in maintaining HIV latency, such as epigenetic and non-epigenetic mechanisms have been discovered, but several obstacles still remain. First and foremost, successful utilization of LRAs requires reactivation of the entire latent HIV provirus pools in different anatomical sites. However, currently, this has not been possible, conceivably because of multiple factors, including suboptimal efficacy of LRAs or the multifactorial nature of latency in different resting memory CD4+ T-cell subsets. In order to overcome these problems, there is a need to use a multifactorial approach involving the use of LRAs from different classes in combinations. Although several studies have recommended the use of LRAs combinations which induce both NF-κB and P-TEFb in order to potently reactivate the latent provirus pools, this approach still needs further indepth study. In this case, instead of using LRAs of different classes but belonging to the same category, such as HDACi only or PKC agonists only, we propose that a combination of LRAs that employ both epigenetic and non-epigenetic mechanisms of HIV latency reactivation should be employed. For instance, increased reactivation of latent HIV-1 has been obtained by using combinations of HDACi with a PKC activator such as prostratin in different systems. The use of such combinations has several merits; for instance, when LRAs are used in such different combinations, lower concentrations could be effective, which could also help to eliminate toxicity and side effects of the drugs. Other studies have also demonstrated that the use of such LRAs combinations has the effects of significantly lowering the threshold levels of transcription factors required to reactivate HIV LTR transcription. Secondly, the toxicity that arises from global TCR activation or the use of PKC agonists needs to be tackled. In this case, the use of non-TCR signals such as those from cytokines and TLRs could be employed to activate NF-κB in combination with activators of the P-TEFb and HDACi.

## Data Availability

Not applicable.

## References

[1] Tyagi M., Bukrinsky M. (2012). Human immunodeficiency virus (HIV) latency: The major hurdle in HIV eradication. Mol. Med..

[2] Ho D.D., Neumann A.U., Perelson A.S., Chen W., Leonard J.M., Markowitz M. (1995). Rapid turnover of plasma virions and CD4 lymphocytes in HIV-1 infection. Nature.

[3] Zhou Y., Zhang H., Siliciano J.D., Siliciano R.F. (2005). Kinetics of human immunodeficiency virus type 1 decay following entry into resting CD4^+^ T cells. J. Virol..

[4] Hokello J., Sharma A.L., Dimri M., Tyagi M. (2019). Insights into the HIV Latency and the Role of Cytokines. Pathogens.

[5] Mbonye U., Karn J. (2011). Control of HIV latency by epigenetic and non-epigenetic mechanisms. Curr. HIV Res..

[6] Han Y., Lassen K., Monie D., Sedaghat A.R., Shimoji S., Liu X., Pierson T.C., Margolick J.B., Siliciano R.F., Siliciano J.D. (2004). Resting CD4^+^ T cells from human immunodeficiency virus type 1 (HIV-1)-infected individuals carry integrated HIV-1 genomes within actively transcribed host genes. J. Virol..

[7] Verdin E., Paras P., Van Lint C. (1993). Chromatin disruption in the promoter of human immunodeficiency virus type 1 during transcriptional activation. EMBO J..

[8] Van Lint C., Emiliani S., Ott M., Verdin E. (1996). Transcriptional activation and chromatin remodeling of the HIV-1 promoter in response to histone acetylation. EMBO J..

[9] Mahmoudi T., Parra M., Vries R.G., Kauder S.E., Verrijzer C.P., Ott M., Verdin E. (2006). The SWI/SNF chromatin-remodeling complex is a cofactor for Tat transactivation of the HIV promoter. J. Biol. Chem..

[10] Coull J.J., Romerio F., Sun J.M., Volker J.L., Galvin K.M., Davie J.R., Shi Y., Hansen U., Margolis D.M. (2000). The human factors YY1 and LSF repress the human immunodeficiency virus type 1 long terminal repeat via recruitment of histone deacetylase 1. J. Virol..

[11] Friedman J., Cho W.K., Chu C.K., Keedy K.S., Archin N.M., Margolis D.M., Karn J. (2011). Epigenetic silencing of HIV-1 by the histone H3 lysine 27 methyltransferase enhancer of Zeste 2. J. Virol..

[12] Blazkova J., Trejbalova K., Gondois-Rey F., Halfon P., Philibert P., Guiguen A., Verdin E., Olive D., Van Lint C., Hejnar J. (2009). CpG methylation controls reactivation of HIV from latency. PLoS Pathog..

[13] Kauder S.E., Bosque A., Lindqvist A., Planelles V., Verdin E. (2009). Epigenetic regulation of HIV-1 latency by cytosine methylation. PLoS Pathog..

[14] Keedy K.S., Archin N.M., Gates A.T., Espeseth A., Hazuda D.J., Margolis D.M. (2009). A limited group of class I histone deacetylases acts to repress human immunodeficiency virus type 1 expression. J. Virol..

[15] Margolis D.M., Somasundaran M., Green M.R. (1994). Human transcription factor YY1 represses human immunodeficiency virus type 1 transcription and virion production. J. Virol..

[16] Williams S.A., Chen L.F., Kwon H., Ruiz-Jarabo C.M., Verdin E., Greene W.C. (2006). NF-kappaB p50 promotes HIV latency through HDAC recruitment and repression of transcriptional initiation. EMBO J..

[17] Tyagi M., Pearson R.J., Karn J. (2010). Establishment of HIV latency in primary CD4^+^ cells is due to epigenetic transcriptional silencing and P-TEFb restriction. J. Virol..

[18] Tyagi M., Karn J. (2007). CBF-1 promotes transcriptional silencing during the establishment of HIV-1 latency. EMBO J..

[19] Sharma A.L., Hokello J., Sonti S., Zicari S., Sun L., Alqatawni A., Bukrinsky M., Simon G., Chauhan A., Daniel R. (2020). CBF-1 Promotes the Establishment and Maintenance of HIV Latency by Recruiting Polycomb Repressive Complexes, PRC1 and PRC2, at HIV LTR. Viruses.

[20] Pearson R., Kim Y.K., Hokello J., Lassen K., Friedman J., Tyagi M., Karn J. (2008). Epigenetic silencing of human immunodeficiency virus (HIV) transcription by formation of restrictive chromatin structures at the viral long terminal repeat drives the progressive entry of HIV into latency. J. Virol..

[21] Marban C., Suzanne S., Dequiedt F., de Walque S., Redel L., Van Lint C., Aunis D., Rohr O. (2007). Recruitment of chromatin-modifying enzymes by CTIP2 promotes HIV-1 transcriptional silencing. EMBO J..

[22] Du Chene I., Basyuk E., Lin Y.L., Triboulet R., Knezevich A., Chable-Bessia C., Mettling C., Baillat V., Reynes J., Corbeau P. (2007). Suv39H1 and HP1gamma are responsible for chromatin-mediated HIV-1 transcriptional silencing and post-integration latency. EMBO J..

[23] Imai K., Togami H., Okamoto T. (2010). Involvement of histone H3 lysine 9 (H3K9) methyltransferase G9a in the maintenance of HIV-1 latency and its reactivation by BIX01294. J. Biol. Chem..

[24] Trushin S.A., Bren G.D., Asin S., Pennington K.N., Paya C.V., Badley A.D. (2005). Human immunodeficiency virus reactivation by phorbol esters or T-cell receptor ligation requires both PKCalpha and PKCtheta. J. Virol..

[25] Williams S.A., Greene W.C. (2007). Regulation of HIV-1 latency by T-cell activation. Cytokine.

[26] Kim Y.K., Mbonye U., Hokello J., Karn J. (2011). T-cell receptor signaling enhances transcriptional elongation from latent HIV proviruses by activating P-TEFb through an ERK-dependent pathway. J. Mol. Biol..

[27] Hokello J., Sharma A.L., Tyagi M. (2020). Efficient Non-Epigenetic Activation of HIV Latency through the T-Cell Receptor Signalosome. Viruses.

[28] Chun T.W., Engel D., Mizell S.B., Ehler L.A., Fauci A.S. (1998). Induction of HIV-1 replication in latently infected CD4^+^ T cells using a combination of cytokines. J. Exp. Med..

[29] Lusic M., Marcello A., Cereseto A., Giacca M. (2003). Regulation of HIV-1 gene expression by histone acetylation and factor recruitment at the LTR promoter. EMBO J..

[30] Benkirane M., Chun R.F., Xiao H., Ogryzko V.V., Howard B.H., Nakatani Y., Jeang K.T. (1998). Activation of integrated provirus requires histone acetyltransferase. p300 and P/CAF are coactivators for HIV-1 Tat. J. Biol. Chem..

[31] Hottiger M.O., Nabel G.J. (1998). Interaction of human immunodeficiency virus type 1 Tat with the transcriptional coactivators p300 and CREB binding protein. J. Virol..

[32] Marzio G., Tyagi M., Gutierrez M.I., Giacca M. (1998). HIV-1 tat transactivator recruits p300 and CREB-binding protein histone acetyltransferases to the viral promoter. Proc. Natl. Acad. Sci. USA.

[33] Easley R., Van Duyne R., Coley W., Guendel I., Dadgar S., Kehn-Hall K., Kashanchi F. (2010). Chromatin dynamics associated with HIV-1 Tat-activated transcription. Biochim. Biophys. Acta.

[34] Agbottah E., Deng L., Dannenberg L.O., Pumfery A., Kashanchi F. (2006). Effect of SWI/SNF chromatin remodeling complex on HIV-1 Tat activated transcription. Retrovirology.

[35] Pumfery A., Deng L., Maddukuri A., de la Fuente C., Li H., Wade J.D., Lambert P., Kumar A., Kashanchi F. (2003). Chromatin remodeling and modification during HIV-1 Tat-activated transcription. Curr. HIV Res..

[36] Hokello J., Sharma A.L., Tyagi M. (2021). AP-1 and NF-kappaB synergize to transcriptionally activate latent HIV upon T-cell receptor activation. FEBS Lett..

[37] West M.J., Lowe A.D., Karn J. (2001). Activation of human immunodeficiency virus transcription in T cells revisited: NF-kappaB p65 stimulates transcriptional elongation. J. Virol..

[38] Tyagi M., Kashanchi F. (2012). New and novel intrinsic host repressive factors against HIV-1: PAF1 complex, HERC5 and others. Retrovirology.

[39] Tyagi S., Ochem A., Tyagi M. (2011). DNA-dependent protein kinase interacts functionally with the RNA polymerase II complex recruited at the human immunodeficiency virus (HIV) long terminal repeat and plays an important role in HIV gene expression. J. Gen. Virol..

[40] Zicari S., Sharma A.L., Sahu G., Dubrovsky L., Sun L., Yue H., Jada T., Ochem A., Simon G., Bukrinsky M. (2020). DNA dependent protein kinase (DNA-PK) enhances HIV transcription by promoting RNA polymerase II activity and recruitment of transcription machinery at HIV LTR. Oncotarget.

[41] Karin M. (1999). How NF-kappaB is activated: The role of the IkappaB kinase (IKK) complex. Oncogene.

[42] Serfling E., Berberich-Siebelt F., Avots A., Chuvpilo S., Klein-Hessling S., Jha M.K., Kondo E., Pagel P., Schulze-Luehrmann J., Palmetshofer A. (2004). NFAT and NF-kappaB factors-the distant relatives. Int. J. Biochem. Cell Biol..

[43] Hayden M.S., Ghosh S. (2004). Signaling to NF-kappaB. Genes Dev..

[44] Giffin M.J., Stroud J.C., Bates D.L., von Koenig K.D., Hardin J., Chen L. (2003). Structure of NFAT1 bound as a dimer to the HIV-1 LTR kappa B element. Nat. Struct. Biol..

[45] Macedo A.B., Novis C.L., De Assis C.M., Sorensen E.S., Moszczynski P., Huang S.H., Ren Y., Spivak A.M., Jones R.B., Planelles V. (2018). Dual TLR2 and TLR7 agonists as HIV latency-reversing agents. JCI Insight.

[46] Mitchell S., Vargas J., Hoffmann A. (2016). Signaling via the NFkappaB system. Wiley Interdiscip. Rev. Syst. Biol. Med..

[47] Verstrepen L., Bekaert T., Chau T.L., Tavernier J., Chariot A., Beyaert R. (2008). TLR-4, IL-1R and TNF-R signaling to NF-kappaB: Variations on a common theme. Cell. Mol. Life Sci. CMLS.

[48] Darcis G., Kula A., Bouchat S., Fujinaga K., Corazza F., Ait-Ammar A., Delacourt N., Melard A., Kabeya K., Vanhulle C. (2015). An In-Depth Comparison of Latency-Reversing Agent Combinations in Various In Vitro and Ex Vivo HIV-1 Latency Models Identified Bryostatin-1+JQ1 and Ingenol-B+JQ1 to Potently Reactivate Viral Gene Expression. PLoS Pathog..

[49] Peterlin B.M., Price D.H. (2006). Controlling the elongation phase of transcription with P-TEFb. Mol. Cell.

[50] Yang Z., Zhu Q., Luo K., Zhou Q. (2001). The 7SK small nuclear RNA inhibits the CDK9/cyclin T1 kinase to control transcription. Nature.

[51] Nguyen V.T., Kiss T., Michels A.A., Bensaude O. (2001). 7SK small nuclear RNA binds to and inhibits the activity of CDK9/cyclin T complexes. Nature.

[52] Barboric M., Lenasi T., Chen H., Johansen E.B., Guo S., Peterlin B.M. (2009). 7SK snRNP/P-TEFb couples transcription elongation with alternative splicing and is essential for vertebrate development. Proc. Natl. Acad. Sci. USA.

[53] Diribarne G., Bensaude O. (2009). 7SK RNA, a non-coding RNA regulating P-TEFb, a general transcription factor. RNA Biol..

[54] He N., Jahchan N.S., Hong E., Li Q., Bayfield M.A., Maraia R.J., Luo K., Zhou Q. (2008). A La-related protein modulates 7SK snRNP integrity to suppress P-TEFb-dependent transcriptional elongation and tumorigenesis. Mol. Cell.

[55] Krueger B.J., Jeronimo C., Roy B.B., Bouchard A., Barrandon C., Byers S.A., Searcey C.E., Cooper J.J., Bensaude O., Cohen E.A. (2008). LARP7 is a stable component of the 7SK snRNP while P-TEFb, HEXIM1 and hnRNP A1 are reversibly associated. Nucleic Acids Res..

[56] Michels A.A., Fraldi A., Li Q., Adamson T.E., Bonnet F., Nguyen V.T., Sedore S.C., Price J.P., Price D.H., Lania L. (2004). Binding of the 7SK snRNA turns the HEXIM1 protein into a P-TEFb (CDK9/cyclin T) inhibitor. EMBO J..

[57] Yik J.H., Chen R., Nishimura R., Jennings J.L., Link A.J., Zhou Q. (2003). Inhibition of P-TEFb (CDK9/Cyclin T) kinase and RNA polymerase II transcription by the coordinated actions of HEXIM1 and 7SK snRNA. Mol. Cell.

[58] Yik J.H., Chen R., Pezda A.C., Samford C.S., Zhou Q. (2004). A human immunodeficiency virus type 1 Tat-like arginine-rich RNA-binding domain is essential for HEXIM1 to inhibit RNA polymerase II transcription through 7SK snRNA-mediated inactivation of P-TEFb. Mol. Cell. Biol..

[59] Tyagi M., Rusnati M., Presta M., Giacca M. (2001). Internalization of HIV-1 tat requires cell surface heparan sulfate proteoglycans. J. Biol. Chem..

[60] Zhou Q., Li T., Price D.H. (2012). RNA polymerase II elongation control. Annu. Rev. Biochem..

[61] Ni Z., Saunders A., Fuda N.J., Yao J., Suarez J.R., Webb W.W., Lis J.T. (2008). P-TEFb is critical for the maturation of RNA polymerase II into productive elongation in vivo. Mol. Cell. Biol..

[62] Lu X., Zhu X., Li Y., Liu M., Yu B., Wang Y., Rao M., Yang H., Zhou K., Chen Y. (2016). Multiple P-TEFbs cooperatively regulate the release of promoter-proximally paused RNA polymerase II. Nucleic Acids Res..

[63] Jang M.K., Mochizuki K., Zhou M., Jeong H.S., Brady J.N., Ozato K. (2005). The bromodomain protein Brd4 is a positive regulatory component of P-TEFb and stimulates RNA polymerase II-dependent transcription. Mol. Cell.

[64] Yang Z., Yik J.H., Chen R., He N., Jang M.K., Ozato K., Zhou Q. (2005). Recruitment of P-TEFb for stimulation of transcriptional elongation by the bromodomain protein Brd4. Mol. Cell.

[65] Barboric M., Yik J.H., Czudnochowski N., Yang Z., Chen R., Contreras X., Geyer M., Matija Peterlin B., Zhou Q. (2007). Tat competes with HEXIM1 to increase the active pool of P-TEFb for HIV-1 transcription. Nucleic Acids Res..

[66] Sedore S.C., Byers S.A., Biglione S., Price J.P., Maury W.J., Price D.H. (2007). Manipulation of P-TEFb control machinery by HIV: Recruitment of P-TEFb from the large form by Tat and binding of HEXIM1 to TAR. Nucleic Acids Res..

[67] Krueger B.J., Varzavand K., Cooper J.J., Price D.H. (2010). The mechanism of release of P-TEFb and HEXIM1 from the 7SK snRNP by viral and cellular activators includes a conformational change in 7SK. PLoS ONE.

[68] Cho S., Schroeder S., Kaehlcke K., Kwon H.S., Pedal A., Herker E., Schnoelzer M., Ott M. (2009). Acetylation of cyclin T1 regulates the equilibrium between active and inactive P-TEFb in cells. EMBO J..

[69] Ramakrishnan R., Dow E.C., Rice A.P. (2009). Characterization of Cdk9 T-loop phosphorylation in resting and activated CD4(+) T lymphocytes. J. Leukoc. Biol..

[70] Sung T.L., Rice A.P. (2009). miR-198 inhibits HIV-1 gene expression and replication in monocytes and its mechanism of action appears to involve repression of cyclin T1. PLoS Pathog..

[71] Dawson M.A., Prinjha R.K., Dittmann A., Giotopoulos G., Bantscheff M., Chan W.I., Robson S.C., Chung C.W., Hopf C., Savitski M.M. (2011). Inhibition of BET recruitment to chromatin as an effective treatment for MLL-fusion leukaemia. Nature.

[72] Mertz J.A., Conery A.R., Bryant B.M., Sandy P., Balasubramanian S., Mele D.A., Bergeron L., Sims R.J. (2011). Targeting MYC dependence in cancer by inhibiting BET bromodomains. Proc. Natl. Acad. Sci. USA.

[73] Delmore J.E., Issa G.C., Lemieux M.E., Rahl P.B., Shi J., Jacobs H.M., Kastritis E., Gilpatrick T., Paranal R.M., Qi J. (2011). BET bromodomain inhibition as a therapeutic strategy to target c-Myc. Cell.

[74] Liu P., Xiang Y., Fujinaga K., Bartholomeeusen K., Nilson K.A., Price D.H., Peterlin B.M. (2014). Release of positive transcription elongation factor b (P-TEFb) from 7SK small nuclear ribonucleoprotein (snRNP) activates hexamethylene bisacetamide-inducible protein (HEXIM1) transcription. J. Biol. Chem..

[75] Richman D.D., Margolis D.M., Delaney M., Greene W.C., Hazuda D., Pomerantz R.J. (2009). The challenge of finding a cure for HIV infection. Science.

[76] Choudhary S.K., Margolis D.M. (2011). Curing HIV: Pharmacologic approaches to target HIV-1 latency. Annu. Rev. Pharmacol. Toxicol..

[77] Xing S., Siliciano R.F. (2013). Targeting HIV latency: Pharmacologic strategies toward eradication. Drug Discov. Today.

[78] Margolis D.M. (2014). How Might We Cure HIV?. Curr. Infect. Dis. Rep..

[79] Martin A.R., Siliciano R.F. (2016). Progress Toward HIV Eradication: Case Reports, Current Efforts, and the Challenges Associated with Cure. Annu. Rev. Med..

[80] Archin N.M., Sung J.M., Garrido C., Soriano-Sarabia N., Margolis D.M. (2014). Eradicating HIV-1 infection: Seeking to clear a persistent pathogen. Nat. Rev. Microbiol..

[81] Deeks S.G. (2012). HIV: Shock and kill. Nature.

[82] Delagreverie H.M., Delaugerre C., Lewin S.R., Deeks S.G., Li J.Z. (2016). Ongoing Clinical Trials of Human Immunodeficiency Virus Latency-Reversing and Immunomodulatory Agents. Open Forum Infect. Dis..

[83] Spivak A.M., Planelles V. (2016). HIV-1 Eradication: Early Trials (and Tribulations). Trends Mol. Med..

[84] Cillo A.R., Sobolewski M.D., Bosch R.J., Fyne E., Piatak M., Coffin J.M., Mellors J.W. (2014). Quantification of HIV-1 latency reversal in resting CD4^+^ T cells from patients on suppressive antiretroviral therapy. Proc. Natl. Acad. Sci. USA.

[85] Spivak A.M., Planelles V. (2018). Novel Latency Reversal Agents for HIV-1 Cure. Annu. Rev. Med..

[86] Bullen C.K., Laird G.M., Durand C.M., Siliciano J.D., Siliciano R.F. (2014). New ex vivo approaches distinguish effective and ineffective single agents for reversing HIV-1 latency in vivo. Nat. Med..

[87] Jiang G., Mendes E.A., Kaiser P., Wong D.P., Tang Y., Cai I., Fenton A., Melcher G.P., Hildreth J.E., Thompson G.R. (2015). Synergistic Reactivation of Latent HIV Expression by Ingenol-3-Angelate, PEP005, Targeted NF-kB Signaling in Combination with JQ1 Induced p-TEFb Activation. PLoS Pathog..

[88] Ait-Ammar A., Kula A., Darcis G., Verdikt R., De Wit S., Gautier V., Mallon P.W.G., Marcello A., Rohr O., Van Lint C. (2019). Current Status of Latency Reversing Agents Facing the Heterogeneity of HIV-1 Cellular and Tissue Reservoirs. Front. Microbiol..

[89] Sherrill-Mix S., Lewinski M.K., Famiglietti M., Bosque A., Malani N., Ocwieja K.E., Berry C.C., Looney D., Shan L., Agosto L.M. (2013). HIV latency and integration site placement in five cell-based models. Retrovirology.

[90] Pardons M., Fromentin R., Pagliuzza A., Routy J.P., Chomont N. (2019). Latency-Reversing Agents Induce Differential Responses in Distinct Memory CD4 T Cell Subsets in Individuals on Antiretroviral Therapy. Cell Rep..

[91] Grau-Exposito J., Luque-Ballesteros L., Navarro J., Curran A., Burgos J., Ribera E., Torrella A., Planas B., Badia R., Martin-Castillo M. (2019). Latency reversal agents affect differently the latent reservoir present in distinct CD4^+^ T subpopulations. PLoS Pathog..

